# KDM3A-mediated SP1 activates PFKFB4 transcription to promote aerobic glycolysis in osteosarcoma and augment tumor development

**DOI:** 10.1186/s12885-022-09636-8

**Published:** 2022-05-19

**Authors:** Wei Wang, Bin Wang

**Affiliations:** Department of Orthopedics, Shengjing Hospital of China Medical University, No. 36, Sanhao Street, Heping District, Shenyang, 110000 Liaoning P.R. China

**Keywords:** KDM3A, SP1, PFKFB4, Osteosarcoma, Glycolysis

## Abstract

**Background:**

Lysine-specific histone demethylase 3A (KDM3A) is a potent histone modifier that is frequently implicated in the progression of several malignancies. However, its role in aerobic glycolysis of osteosarcoma (OS) remains unclear.

**Methods:**

KDM3A expression in OS tissues was determined by immunohistochemistry, and that in acquired OS cells was determined by RT-qPCR and western blot assays. KDM3A was silenced in OS cells to examine cellular behaviors and the aerobic glycolysis. Stably transfected cells were injected into nude mice for in vivo experiments. The downstream targets of KDM3A were predicted by bioinformatics systems and validated by ChIP-qPCR. Rescue experiments of SP1 and PFKFB4 were performed to examine their roles in the KDM3A-mediated events.

**Results:**

KDM3A was highly expressed in OS tissues and cells. Knockdown of KDM3A weakened OS cell growth and metastasis in vivo and in vitro, and it suppressed the aerobic glycolysis in OS cells. KDM3A enhanced the transcription of SP1 by demethylating H3K9me2 on its promoter. Restoration of SP1 rescued growth and metastasis of OS cells and recovered the glycolytic flux in cells suppressed by knockdown of KDM3A. SP1 bound to the PFKFB4 promoter to activate its transcription and expression. PFKFB4 expression in OS cells was suppressed by KDM3A silencing but increased after SP1 restoration. Overexpression of PFKFB4 significantly promoted OS cell growth and metastasis as well as the glycolytic flux in cells.

**Conclusion:**

This paper elucidates that upregulation of PFKFB4 mediated by the KDM3A-SP1 axis promotes aerobic glycolysis in OS and augments tumor development.

**Supplementary Information:**

The online version contains supplementary material available at 10.1186/s12885-022-09636-8.

## Background

Osteosarcoma (OS), the most common and aggressive bone tumor predominantly affecting children, adolescents, and young adults, is characterized by frequent recurrence and metastasis [1]. The annual incidence of OS in the general population is 2–3 per million, peaking at 8–11 per million in adolescents aged 15–19 years [2]. OS  more frequently develops in the long bones such as the femur, tibia, and humerus, and less frequently in the skull, jaw, and pelvis [3]. Therapy must include the complete surgical resection of all detectable tumor sites and multiagent chemotherapy, which should include all or several of the following four drugs: doxorubicin (adriamycin), cisplatin, high-dose methotrexate, and ifosfamide [2, 4]. However, although the patients with surgically resectable disease may survive for a long time, the prognosis of patients with recurrent and unresectable disease is always poor [5, 6]. Moreover, the poor prognosis of OS has not improved over the past three decades [7]. New therapeutic methods are urgently needed.

Uncontrolled proliferation of cells is the most universal and well-recognized feature of cancer, and aberrant energy metabolism plays a considerable role in the maintenance of massive cell growth [8, 9]. Cancer cells tend to undergo aerobic glycolysis instead of mitochondrial oxidative phosphorylation for energy generation, as manifested by significantly elevated glucose uptake and lactate production, termed “Warburg Effect” [10]. Targeting molecules or enzymes involved in the metabolic shift, therefore, has been proposed as a therapeutic option for the control of malignancies, including OS [11, 12].

In eukaryotes, histone post-translational modifications, including methylation, ubiquitination and acetylation, affect DNA accessibility and control gene expression, therefore participating in the epigenetic regulation of an array of diseases [13]. Lysine-specific histone demethylase 3A (KDM3A), also known as JMJD1, can catalyze the demethylation of mono- and di-methylated histone H3 lysine 9 (H3K9me1/me2), which are transcriptionally repressive markers, therefore regulating transcriptional activation [14]. This histone demethylase has been frequently correlated with cancer progression and therefore proposed as a potential target for anti-cancer therapy [15]. Interestingly, KDM3A has been reported to epigenetically activate melanoma cell adhesion molecules to promote migration and metastasis of Ewing Sarcoma, the second most frequent solid pediatric tumor of bone and soft tissue [16]. However, its role in OS has not been investigated yet.

In this research, integrated bioinformatics analyses using the advanced analysis tools and systems suggested the Sp1 transcription factor (SP1) as a candidate target of KDM3A, and 6-phosphofructo-2-kinase/fructose-2,6-biphosphatase 4 (PFKFB4) as a transcriptional target of SP1. SP1 is a nuclear transcription factor participating in many cellular processes, such as differentiation, proliferation and apoptosis [17], and it is highly expressed in many cancers and usually correlated with poor prognosis [18]. PFKFB4 mediates glycolysis by maintaining the levels of fructose-2,6-bisphosphate (F2,6-BP), a key allosteric activator of the rate-limiting enzyme 6-phosphofructo-1-kinase [19]. Therefore, we hypothesized that there might be a KDM3A/SP1/PFKFB4 axis involved in glycolysis and development of OS.

## Methods

### Clinical samples

Forty-two OS patients who underwent surgery in Shengjing Hospital of China Medical University from September 2018 to June 2020 were enrolled in the present research. The primary tumor and peritumor bone tissues were collected during surgery and stored at − 80 °C. All patients had complete clinical information and were free of other malignancies. A signed informed consent form was received from each enrolled patient. Primary tumor tissues were collected from the areas with abundant live tumor cells rather than the areas with severe necrosis and bleeding. Peritumor tissues were obtained at 3–5 cm adjacent to the tumor sites. All collected tissues were confirmed by experienced pathologists. Formalin-fixed paraffin-embedded tissue (FFPET) samples were used for immunohistochemistry (IHC) to analyze protein expression. All procedures were ratified by the Ethics Committee of Shengjing Hospital of China Medical University and conducted in accordance with the *Declaration of Helsinki.*

### IHC

FFPET samples were cut into 5-μm sections, dewaxed in xylene and rehydrated in a descending series of alcohol (100, 95 and 75%). After that, the tissue sections were boiled in ethylene diamine tetraacetic acid for 20 min, cooled for 10 min, and rinsed with phosphate-buffered saline (PBS). After incubation with the primary antibodies against KDM3A (1:200, 12835–1-AP, Proteintech Group, Wuhan, Hubei, China), SP1 (1:500, ab231778, Abcam Inc., Cambridge, MA, USA) and PFKFB4 (1:100, ab137785, Abcam) at 37 °C for 2 h, the tissue slides were further incubated with goat anti-rabbit IgG H&L (HRP) (1:2000, ab205718, Abcam) for 30 min. A 3,3′-diaminobenzidine substrate kit (Abcam) was used for color development, and hematoxylin was used for counter-staining of nuclei. After that, the tissue sections were dehydrated, cleared and sealed for observation under a microscope (Carl Zeiss, Oberkochen, Germany).

The staining was scored by three pathologists who had no idea of the tissue types. The score for the portion of positively-stained cells was as follows: < 5% (0); 5–25% (1); 25–50% (2); 50–75% (3) and > 75% (4). The score for staining intensity was as follows: no staining (0); light brown (1); brown (2), and dark brown (3). The product of the two scores was calculated as the final IHC score (0 ~ 12).

### Cell culture and transfection

Immortalized human fetal osteoblasts hFOB1.19 (CRL-11372) were procured from American Type Culture Collection. Cells were cultured in the medium mixed by Ham’s F12 Medium and Dulbecco’s modified Eagle’s medium (1:1; all provided by Thermo Fisher Scientific Inc., Waltham, MA, USA), which was supplemented with 2.5 mM L-glutamine, 0.3 mg/mL G418 and 10% fetal bovine serum (FBS). An immortalized human osteoprecursor cell line OPC1 (T0230) was procured from ABM Inc. (Zhenjiang, Jiangsu, China). Cells were cultured in the Prigrow VIII medium (TM018; ABM Inc.) containing 5% FBS, 750 U/mL human A/D interferon, 0.2 mg/mL G418, and 1% penicillin-streptomycin. The culture condition for both cells was 37 °C with 5% CO_2_.

Human OS cell lines MG63 (CL-0157), Saos-2 (CL-0202), U2OS (CL-0236) and HOS (CL-0360). OS cells were cultured in minimum essential medium (MEM; Procell Life Science & Technology Co., Ltd., Wuhan, Hubei, China) supplemented with 10% FBS and 1% penicillin-streptomycin. The cells were cultured at 37 °C with 5% CO_2_ as well.

Short hairpin (sh) RNA of KDM3A (sh-KDM3A 1, 2, 3#), the DNA overexpression plasmids pCAG-SP1 and pCAG-PFKFB4 and the negative control (NC) were all procured from VectorBuilder Company (Guangzhou, Guangdong, China). The shRNA or plasmids were transfected into cells according to the instructions of Lipofectamine 3000 (Thermo Fisher Scientific).

### Reverse transcription-quantitative polymerase chain reaction (RT-qPCR)

Total RNA from tissues and cells was isolated using the TRIzol reagent and then reverse-transcribed to complementary DNA (cDNA) using a PrimeScript RT kit with gDNA Eraser (Takara Holdings Inc., Kyoto, Japan). Real-time qPCR was performed on an Applied Biosystems 7500 system (Applied Biosystems, Inc., Carlsbad, CA, USA) according to the instructions of TB Green® Premix Ex Taq™ II (Takara). Relative gene expression was conducted by the 2^-ΔΔCt^ method. The primers are listed in Table 1, where GAPDH was the endogenous loading.

**Table 1:** Primer sequences for RT-qPCR

Gene	Forward primer (5′-3′)	Reverse primer (5′-3′)
KDM3A	GCCAACATTGGAGACCACTTCTG	CTCGAACACCTTTGACAGCTCG
SP1	ACGCTTCACACGTTCGGATGAG	TGACAGGTGGTCACTCCTCATG
PFKFB4	GATCCTGAGGTCATAGCTGCCA	CTATCCAGGTCCTCATCTAGCG
SIN3A	CAGAATGACACCAAGGTCCTGAG	CATACGCAAGTGAGAGGTGTGG
GAPDH	GTCTCCTCTGACTTCAACAGCG	ACCACCCTGTTGCTGTAGCCAA

*RT-qPCR* Reverse transcription quantitative polymerase chain reaction, *KDM3A* Lysine-specific histone demethylase 3A, *SP1* Sp1 transcription factor, *PFKFB4* 6-phosphofructo-2-kinase/fructose-2,6-biphosphatase 4, *SIN3A* SIN3 transcription regulator family member A, *GAPDH* glyceraldehyde-3-phosphate dehydrogenase

### Western blot analysis

Tissues or cells were lysed in radio-immunoprecipitation assay (RIPA) buffer (Beyotime Biotechnology Co., Ltd., Shanghai, China) on ice to extract total protein. The protein sample was quantified using a bicinchoninic acid kit (Thermo Fisher Scientific) and then transferred onto polyvinylidene fluoride membranes after gel electrophoresis. After being blocked in 5% not-fat milk, the membranes were incubated with the primary antibodies (Table 2) at 4 °C overnight, and then with goat anti-rabbit IgG H&L (HRP) (1:10,000, ab205718, Abcam) at ambient temperature for 2 h. An enhanced chemiluminescence kit was used for the development of the protein bands, and the quantification analysis was performed by the Quantity one software. β-actin was used as the endogenous control.

**Table 2:** Primary antibodies for western blot analysis

Antibodies	Dilution	Cat. NO.	Manufacture
KDM3A	1:1000	12,835–1-AP	PTGCN
β-actin	1:1000	ab115777	Abcam
SP1	1:1000	ab231778	Abcam
SIN3A	1:1000	#8056	CST
PFKFB4	1:1000	ab137785	Abcam

*KDM3A* Lysine-specific histone demethylase 3A, *SP1* Sp1 transcription factor, *SIN3A* SIN3 transcription regulator family member A, *PFKFB4* 6-phosphofructo-2-kinase/fructose-2,6-biphosphatase 4

### Cell counting kit-8 (CCK-8) assay

Exponentially growing OS cells were sorted into 96-well plates (1 × 10^4^ cells/well) and cultured for indicated durations (0, 1, 2, 3, 4 days, respectively). Each well was then added with 10 μL CCK-8 solution (Beyotime), followed by two more hours of incubation. The optical density (OD) at 450 nm was examined using a microplate reader (Bio-Rad, Inc., Hercules, CA, USA).

### Colony formation assay

Cells were cultured in 6-well plates at 1000 cells per well at 37 °C with 5% CO_2_. After 2 weeks, the cells were rinsed with PBS, and the cell colonies were fixed by 4% paraformaldehyde (PFA) for 15 min and stained with crystal violet for 15 min. The number of cell colonies was counted under the microscope.

### Scratch test

Cells were sorted in 12-well plates at 5.0 × 10^5^ cells/well. After 24 h when the cell confluence reached approximately 80%, a sterile 200-μL pipette tip was used to make scratches on the cell monolayer along the midline of the wells. The cells were rinsed with medium, and then the culture medium was replaced by serum-free MEM. The width of scratches on the 0 and 24 h was captured and analyzed using the Image J software (NIH, Bethesda, MD, USA).

### Transwell assay

Invasion of cells was examined using the Transwell chambers (8-μm diameter; Corning Glass Works, Corning, NY, USA). The apical chambers were precoated with 50 μL Matrigel (BD Biosciences, Franklin Lakes, NJ, USA) and inserted into the 24-well plates overnight. The OS cells were digested in trypsin and resuspended in serum-free MEM to 2 × 10^5^ cells/mL. The basolateral chambers were supplemented with 250 μL 10% FBS-supplemented MEM, whereas the apical chambers were added with 100 μL OS cell suspension. The plates were placed in a 37 °C incubator for 24 h. After that, the cells invaded into the lower membranes were fixed with 4% PFA for 15 min and stained with crystal violet. The images were captured under the microscope, and the number of invaded cells was counted using the Image J software (NIH).

### Animal experiments

Female BALB/C nude mice (3–4 weeks old) were procured from Vital River Laboratory Animal Technology Co., Ltd. (Beijing, China). The mice were randomly assigned into 6 groups: sh-NC, sh-KDM3A, sh-KDM3A + pCAG-NC, sh-KDM3A + pCAG-SP1, pCAG-NC, and pCAG-PFKFB4, *n* = 10 in each group.

Five mice in each group were used for the tumor growth assay. The HOS cells with corresponding transfection were administrated into the right flank of mice (1 × 10^6^ cells/mouse) via subcutaneous injection. The length and width of the xenograft tumors in mice were examined every 5 days for 30 days. To reduce animal suffering, the experimental period for animals in the pCAG-NC and pCAG-PFKFB4 groups where the tumor grew rapidly, was only 20 days. The volume of tumors was calculated as follows: volume = length × width^2^/2. After the last volume measurement, the mice were euthanized via intraperitoneal injection of 150 mg/kg pentobarbital sodium. The tumors were taken out and weighed.

Another five mice in each group were used for the tumor metastasis assay. The HOS cells were administrated into mice (4 × 10^6^ cells/mouse) via tail vein injection. After 45 days (the period for mice in the pCAG-NC and pCAG-PFKFB4 was 30 days), the mice were euthanized. The lung tissues were collected to examine metastatic lesions. This study was approved by the Animal Ethics Committee of Shengjing Hospital of China Medical University and conforms to the ARRIVE guidelines. All animal procedures adhered to the Guide for the Care and Use of Laboratory Animals (NIH Publication No. 85–23, revision 1996).

### Hematoxylin and eosin (HE) staining

The mouse lung tissues were fixed in 4% PFA overnight, embedded in paraffin, and cut into 5-μm sections. The sections were dewaxed in xylene and rehydrated, and then stained with hematoxylin for 10 min. After that, the sections were differentiated in 0.3% HCl-ethanol for 30 s, and counter-stained with eosin for 2 min. The stained sections were observed under the microscope (Olympus Optical Co., Ltd., Tokyo, Japan). The tumor infiltration area was evaluated using the Image J software.

### Enzyme-linked immunosorbent assay (ELISA)

ELISA kits procured from BioVision (Milpitas, CA, USA) were used to examine the glycolysis-related biomarkers. The glucose uptake was measured using a Glucose Uptake Colorimetric Assay Kit (Catalog #: K676) by examining the OD value at 412 nm. The lactate production in cells was measured using a Lactate Colorimetric Assay Kit (Catalog #: K627) by examining the OD at 450 nm. The production of fructose-6-phosphate (F6P) in cells was measured using a Fructose-6-Phosphate Fluorometric Assay Kit (Catalog #: K689) by examining the fluorescence intensity of Ex/Em (535/587 nm). The production of glyceraldehyde-3-phosphate (G3P) in cells was measured using a G3P Fluorometric Assay Kit (Catalog #: K2018) by examining the fluorescence intensity of Ex/Em (535/587 nm). All procedures were performed according to the instruction manuals.

### Metabolic flux analysis

The oxygen consumption rate (OCR) and extracellular acidification rate (ECAR) in cells were examined using a Seahorse XF Cell Mito Stress Test Kit and a Seahorse XF Glycolysis Stress Test Kit, respectively, on a Seahorse XF-96 metabolic flux analyzer (Seahorse Bioscience, North Billerica, MA, USA). In short, OS cells were loaded onto the XF-96 cell culture microplates at 3 × 10^4^ cells per well. After probe calibration, for OCR, each well was added with 1 mM oligomycin, 1 mM FCCP, 2 mM antimycin A and 2 mM Rotenone (A&R); for ECAR, each well was added with 10 mM glucose, 1 mM oligomycin and 80 mM 2-DG. The data were evaluated and analyzed using the Seahorse XF-96 Wave software.

### Chromatin immunoprecipitation (ChIP)-qPCR

An EZ-Magna ChIP kit (Millipore, Billerica, MA, USA) was used for ChIP assay. In brief, exponentially growing cells (1 × 10^7^) were crosslinked in 1% methanol for 10 min and quenched in glycine at room temperature for 5 min. Cells were treated with RIPA buffer and ultrasonicated to produce 200–1000 bp chromatin fragments. The chromatin fragments were incubated with the ChIP-grade antibodies anti-H3K9me2 (1:100, ab176882, Abcam), anti-KDM3A (1:100, 12835–1-AP, Proteintech) or anti-SP1 (1:200, ab231778, Abcam) overnight. Rabbit IgG (Millipore) was used as control. The chromatin was then eluted with elution buffer, treated with proteinase K, and de-crosslinked. The enrichment of SP1 or PFKFB4 promoters was examined by qPCR. The primer sequences were as follows: SP1 promoter: Forward primer: 5′-TCCACCGTCTTTCTTCTGCA-3′; Reverse primer: 5′-CTGACGAGGCAAGCGAAC-3′. PFKFB4 promoter: Forward primer: 5′- CTTCAGGCCAGGATCGAGAA - 3′; Reverse primer: 5′-GAGGTAGCAGAATTCACGCG-3′.

### Dual-luciferase reporter assay

The putative (wild type, WT) binding site between SP1 and PFKFB4 promoter was obtained from Jaspar (http://jaspar.genereg.net/), and the mutant type (MT) site was designed. The PFKFB4 promoter sequence containing the WT or MT site was inserted into the pGL3-basic vector (Promega Corporation, Madison, WI, USA) to construct luciferase reporter vectors. Well-constructed vectors were co-transfected with either pCAG-SP1 or pCAG-SP1-NC into 293 T (ATCC, Manassas, VA, USA). After 48 h, the luciferase activity in cells was examined using a dual-luciferase activity kit (Promega).

### Statistical analysis

GraphPad Prism 8.02 (GraphPad, La Jolla, CA, USA) was used for data analysis. Measurement data from three independent experiments were expressed as the mean ± standard deviation (SD). Differences were analyzed by the *t* test (two groups) or the one- or two-way analysis of variance (ANOVA) followed by Tukey’s post-hoc test. *p < 0.05 was set as the cut-off value of statistical significance.

## Results

### KDM3A is highly expressed in OS and is correlated with tumor progression in patients

**Fig. 1:**
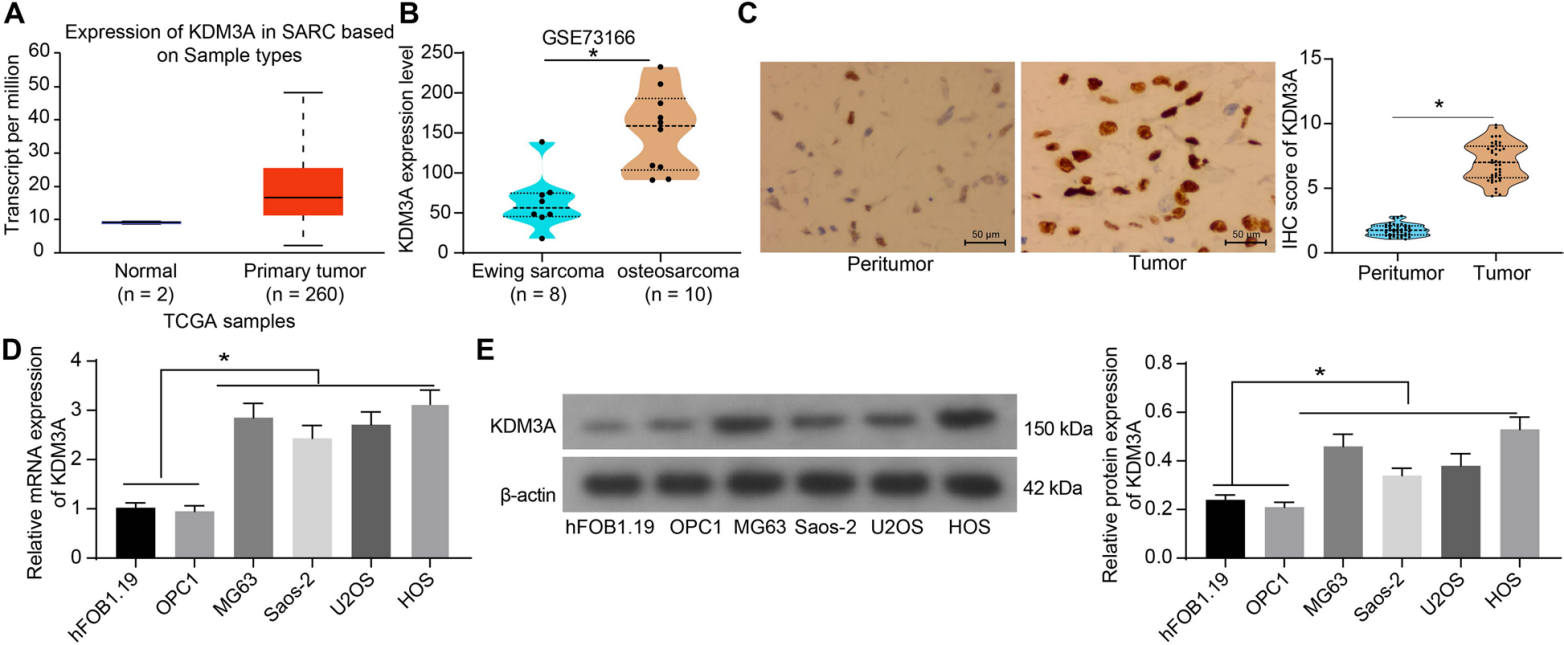
KDM3A is highly expressed in OS and indicates poor prognosis for patients. **A** expression profile of KDM3A in sarcoma in TCGA database; **B** KDM3A expression in Ewing Sarcoma and OS in the GEO GSE73166 dataset; **C** IHC score of KDM3A in the collected tumor and peritumor tissue samples; D-E, KDM3A mRNA (**D**) and protein (**E**) levels in the normal cells (hFOB1.19 and OPC1) and OS cell lines (MG63, Saos-2, U2OS and HOS) detected by RT-qPCR and western blot analysis. Three independent experiments were performed. Data were analyzed by the unpaired *t* test (**B**), paired *t* test (**C**), and one-way ANOVA (**D** and **E**) followed by Tukey’s test. *p < 0.05

Compared to its oncogenic role in Ewing Sarcoma, less is known about the function of KDM3A in OS. According to the data in the UALCAN portal site (http://ualcan.path.uab.edu/index.html) of The Cancer Genome Atlas (TCGA), the KDM3A expression was suggested to be elevated in sarcoma (Fig. 1A). Considering the limited sample size of normal tissues (*n* = 2) in TCGA, we further analyzed a GSE73166 dataset from the Gene Expression Omnibus (GEO) database (https://www.ncbi.nlm.nih.gov/geo/), which suggested that the KDM3A expression was even higher in OS tissue samples than that in Ewing Sarcoma (Fig. 1B). This can indirectly prove that KDM3A has a high-expression pattern in OS.

Thereafter, the KDM3A level in the collected tissue samples was determined by the IHC assay. It was observed that KDM3A was highly expressed in OS tissues versus the normal peritumor bone tissues (Fig. 1C). Based on the IHC scores, the OS patients were allocated into high (*n* = 20) and low (*n* = 22) KDM3A IHC score groups. The analysis on clinicopathologic parameters of patients (Table 3) suggested that a high KDM3A level was correlated with large tumor size, lymph node metastasis, and increased AJCC/TNM stages, although the level was not correlated with the sex, age, and tumor location.

**Table 3:** Correlation between KDM3A IHC score and the clinicopathologic parameters of patients with OS

Parameters		KDM3A IHC score		*P* value
		High (*n* = 20)	Low (*n* = 22)	
Sex	Male (*n* = 27)	12	15	
	Female (*n* = 15)	8	7	0.7488
Age (year)	< 20 (*n* = 31)	17	14	0.1658
	≥20 (*n* = 11)	3	8	
Tumor size (cm)	< 5 (*n* = 19)	4	15	**0.0023
	≥5 (*n* = 23)	16	7	
Location	Upper limb bone (*n* = 17)	10	7	0.3462
	Lower limb bone (*n* = 25)	10	15	
Lymph node metastasis	Positive (*n* = 28)	17	11	*0.0232
	Negative (*n* = 14)	3	11	
AJCC/TNM stage	I ~ II (*n* = 12)	2	10	*0.0167
	III ~ IV (*n* = 30)	18	12	

*KDM3A* Lysine-specific histone demethylase 3A, *IHC* Immunohistochemistry, *OS* Osteosarcoma, *AJCC/TNM* American Joint Committee on Cancer/Tumor node metastasis; *p < 0.05; **p < 0.01

In cells, high KDM3A expression, likewise, was observed in all OS cell lines (MG63, Saos-2, U2OS and HOS) compared to the normal hFOB1.19 and OPC1 cells according to the RT-qPCR and western blot assays (Fig. 1D, E). The MG63 and HOS cells with the most aberrant KDM3A expression were applied in the subsequent tests.

### KDM3A is crucial for the maintenance of the growth and metastasis of OS cells

**Fig. 2:**
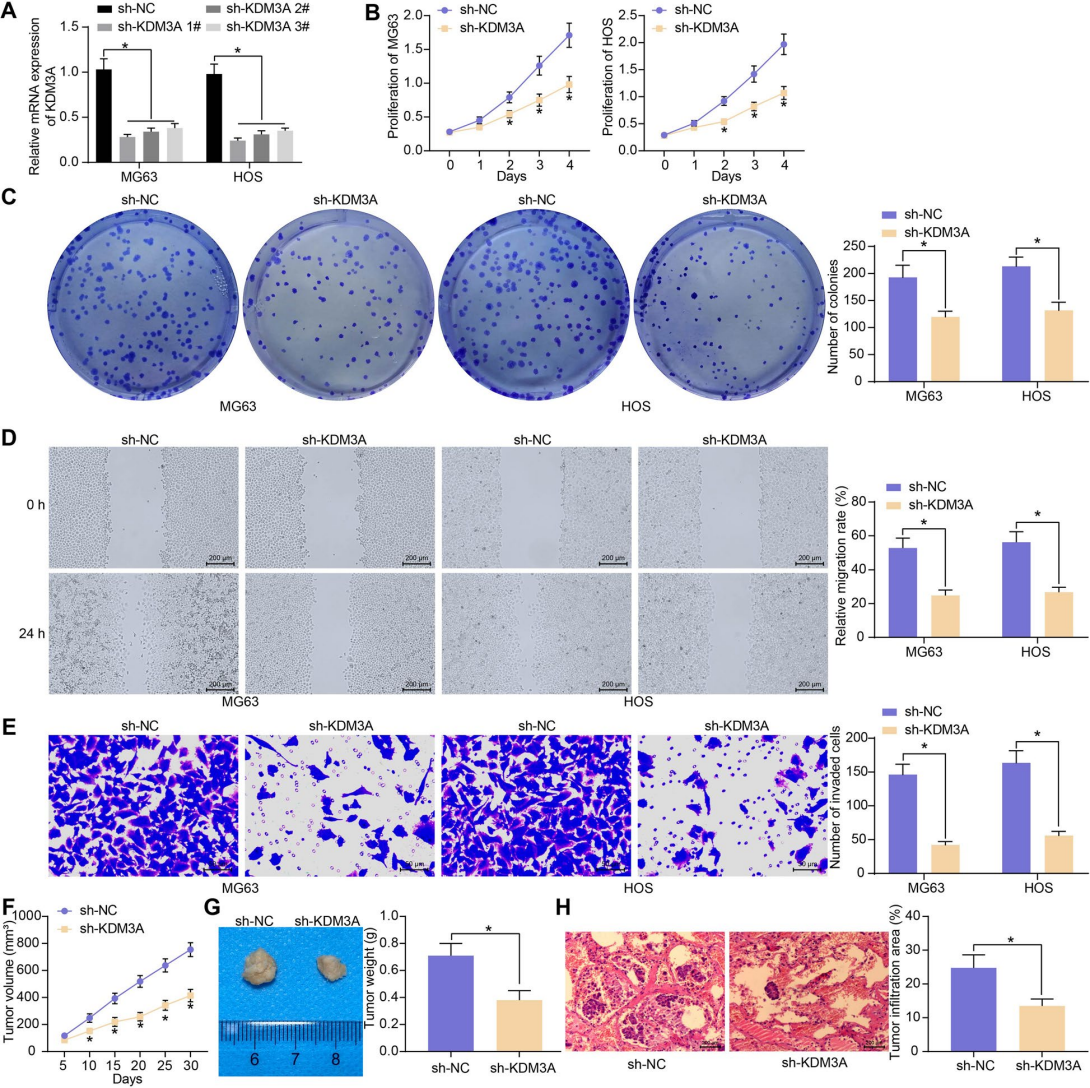
KDM3A is crucial for the maintenance of the growth and metastasis of OS cells. **A** knockdown efficiency of sh-KDM3A 1, 2, 3# in MG63 and HOS cells examined by RT-qPCR; **B** proliferative activity of MG63 and HOS cells detected by the CCK-8 assay; **C** colony formation ability of MG63 and HOS cells examined by the colony formation assay; **D** migration of MG63 and HOS cells examined by the scratch test; **E** invasion of MG63 and HOS cells examined by the Transwell assay; **F** growth rate of xenograft tumors formed by HOS cells in nude mice; **G** weight of the xenograft tumors on day 30; **H** tumor infiltration in mouse lung tissues 45 days after tail vein injection of HOS cells examined by HE staining. For cellular experiments, three repetitions were performed; for animal experiments, *n* = 5 in each group. Data were analyzed by the unpaired *t* test (**G**, **H**) and two-way ANOVA (**A**-**F**) followed by Tukey’s test. *p < 0.05

KDM3A was silenced in MG63 and HOS cells through the transfection of shRNA (sh-KDM3A 1, 2, 3#). The sh-KDM3A1# with the greatest suppressive effect (Fig. 2A) was selected for subsequent use. After KDM3A silencing, the proliferative activity (Fig. 2B) and colony formation ability (Fig. 2C) of the OS cells, as respectively detected by the CCK-8 and colony formation assays, were significantly blocked. The results of scratch tests and Transwell assays indicated that the migration and invasion activities of OS cells were weakened as well following KDM3A silencing (Fig. 2D, E).

Xenograft tumors were induced in nude mice for in vivo tests, where only HOS cells were used to avoid unnecessary animal usage. Similarly, silencing of KDM3A in HOS cells reduced the growth rate of xenograft tumors (Fig. 2F) as well as the tumor weight (Fig. 2G) on day 30. Silencing of KDM3A also reduced the tumor infiltration area in mouse lung areas when the HOS cells were injected via the tail vein (Fig. 2H).

### KDM3A helps maintain the aerobic glycolysis in OS cells

**Fig. 3:**
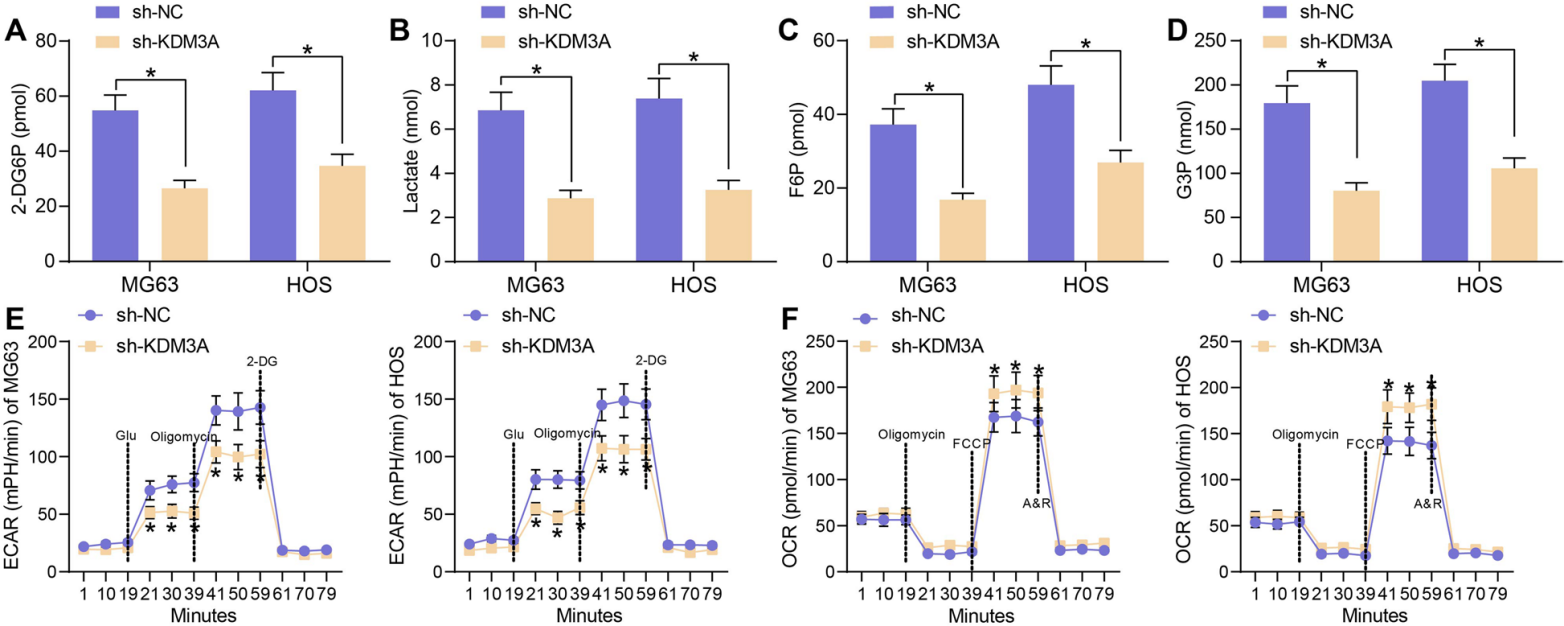
KDM3A helps maintain the aerobic glycolysis in OS cells. The glucose uptake (**A**), lactate production (**B**), F6P production (**C**), G3P production (**D**), ECAR (**E**) and OCR (**F**) in OS cells after KDM3A inhibition. Three independent experiments were performed. Differences were analyzed by the two-way ANOVA (**A**-**F**) followed by Tukey’s test. *p < 0.05

After knockdown of KDM3A, it was noteworthy that the glucose uptake and lactate production in OS cells was significantly suppressed (Fig. 3A, B), along with decreased levels of glycolysis-related intermediate products F6P and G3P in cells (Fig. 3C, D).

The glycolytic activity in cells was further examined by determining the ECAR and OCR in cells. Knockdown of KDM3A significantly reduced the ECAR (Fig. 3E) and accordingly, elevated the OCR in OS cells (Fig. 3F).

### KEM3A mediates H3K9me2 modification on SP1 promoter to regulate its expression

**Fig. 4:**
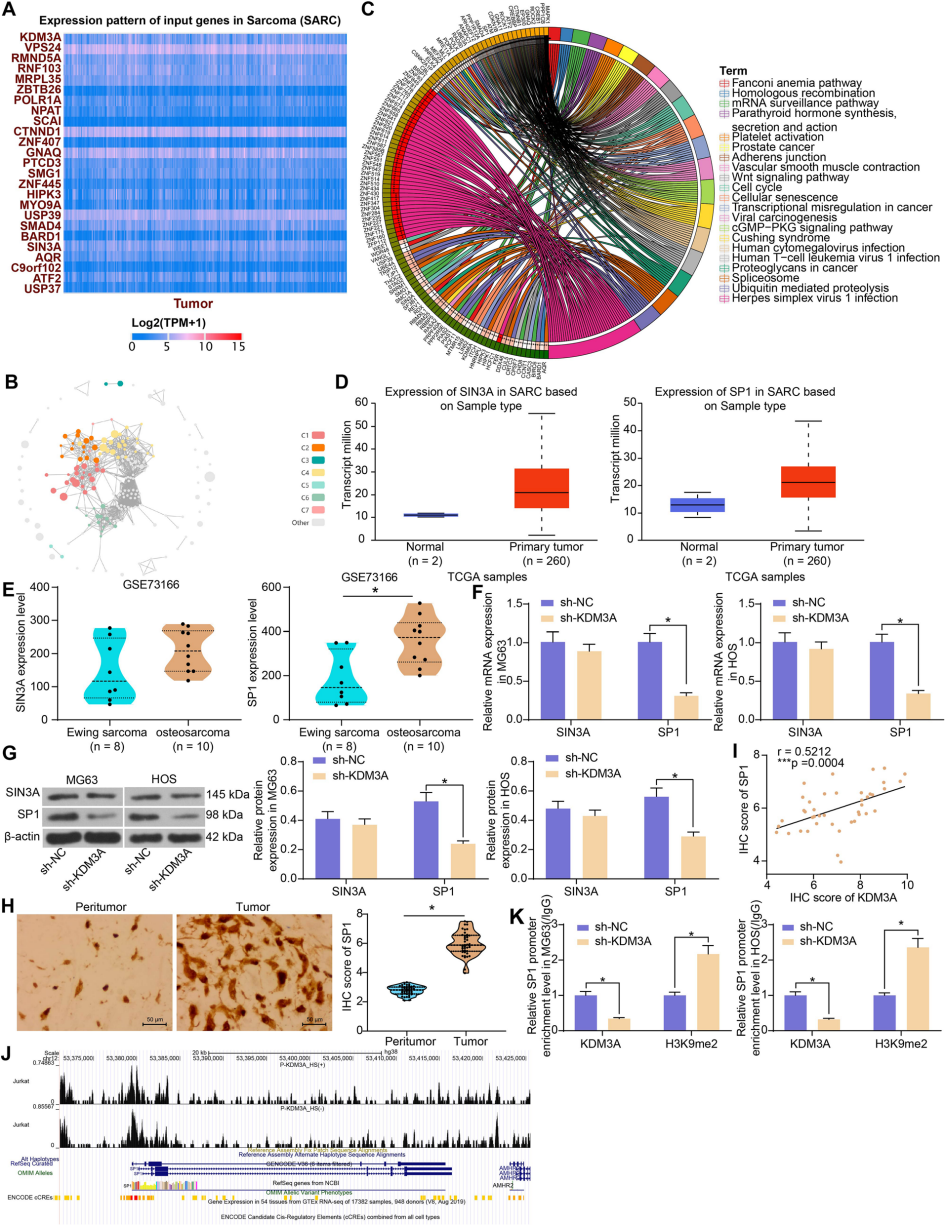
KDM3A mediates H3K9me2 modification on SP1 promoter to regulate its expression. **A** genes positively correlated with KDM3A in sarcoma tissues in UALCAN system; **B**, **C** KEGG pathway enrichment analysis on the candidate target genes of KDM3A; **D** expression profiles of SIN3A and SP1 in sarcoma in the GEPIA database; **E** expression patterns of SIN3A and SP1 in Ewing Sarcoma and OS in the GEO GSE73166 dataset; **F**, **G** mRNA (**F**) and protein (**G**) expression of SIN3A and SP1 in OS cells transfected with sh-KDM3A examined by RT-qPCR and western blot analysis, respectively; **H** IHC score of SP1 in the collected tumor and peritumor bone tissue samples; **I** correlation between KDM3A and SP1 IHC scores in OS tissues; **J** enrichment of KDM3A on SP1 promoter; **K** KDM3A and H3K9me2 modification levels on SP1 promoter in OS cells following KDM3A inhibition examined by the ChIP-qPCR assay. Three independent experiments were performed. Differences were analyzed by the unpaired *t* test (**E**), paired *t* test (**H**), and two-way ANOVA (**F**, **G** and **K**) followed by Tukey’s test. Correlation between variables was analyzed by the Pearson’s correlation analysis (I). *p < 0.05

KDM3A catalyzes the demethylation of transcriptionally repressive H3K9me1/me2 in vitro and in vivo, with a preference for dimethylated residues, thereby regulating transcriptional activation [14, 20]. We therefore predicted genes having positive correlations with KDM3A in sarcoma tissues in the UALCAN system, and those having an over 0.5 correlation coefficient were selected for subsequent use (Fig. 4A). A functional cluster analysis was performed on these genes using the Kyoto Encyclopedia of Genes and Genomes (KEGG) pathway enrichment analysis [21–23] (https://www.kegg.jp/) (Fig. 4B). The enriched pathways with *p* < 0.001 were collected for further analysis (Fig. 4C).

Among the pathways, hsa05202 (Transcriptional misregulation in cancer) attracted our attention since KDM3A mainly functions as a transcriptional regulator in cancer [15]. The expression profiles of the genes predicted above in sarcoma were further explored in UALCAN. SIN3 transcription regulator family member A (SIN3A) and SP1 were suggested to be highly expressed in sarcoma (Fig. 4D). Moreover, the SIN3A and SP1 levels were analyzed in the GSE73166 dataset as well. It was suggested that the SIN3A expression showed no significant difference between Ewing Sarcoma and OS, whereas SP1 was significantly upregulated in OS samples (Fig. 4E). Of note, in OS cells, transfection of KDM3A significantly reduced the expression of SP1 but had little effect on the expression of SIN3A (Fig. 4F, G). Moreover, the IHC assay indicated that SP1 was highly expressed in the OS tissue samples (Fig. 4H), and the IHC score of SP1 showed a positive correlation with KDM3A (Fig. 4I).

Data in the Cistrome Data Browser (http://cistrome.org/db/#/) of the ChIP-seq database suggested that KDM3A had a significantly strengthened binding peak with SP1 promoter sites (chr12: 53,380,045-53,380,245) (Fig. 4J). To validate this, a ChIP-qPCR was performed, suggesting that the silencing of KDM3A in OS cells reduced the level of KDM3A but increased the H3K9me2 modification level on SP1 promoter (Fig. 4K).

### SP1 is essential for the KDM3A-mediated aerobic glycolysis in OS cells

**Fig. 5:**
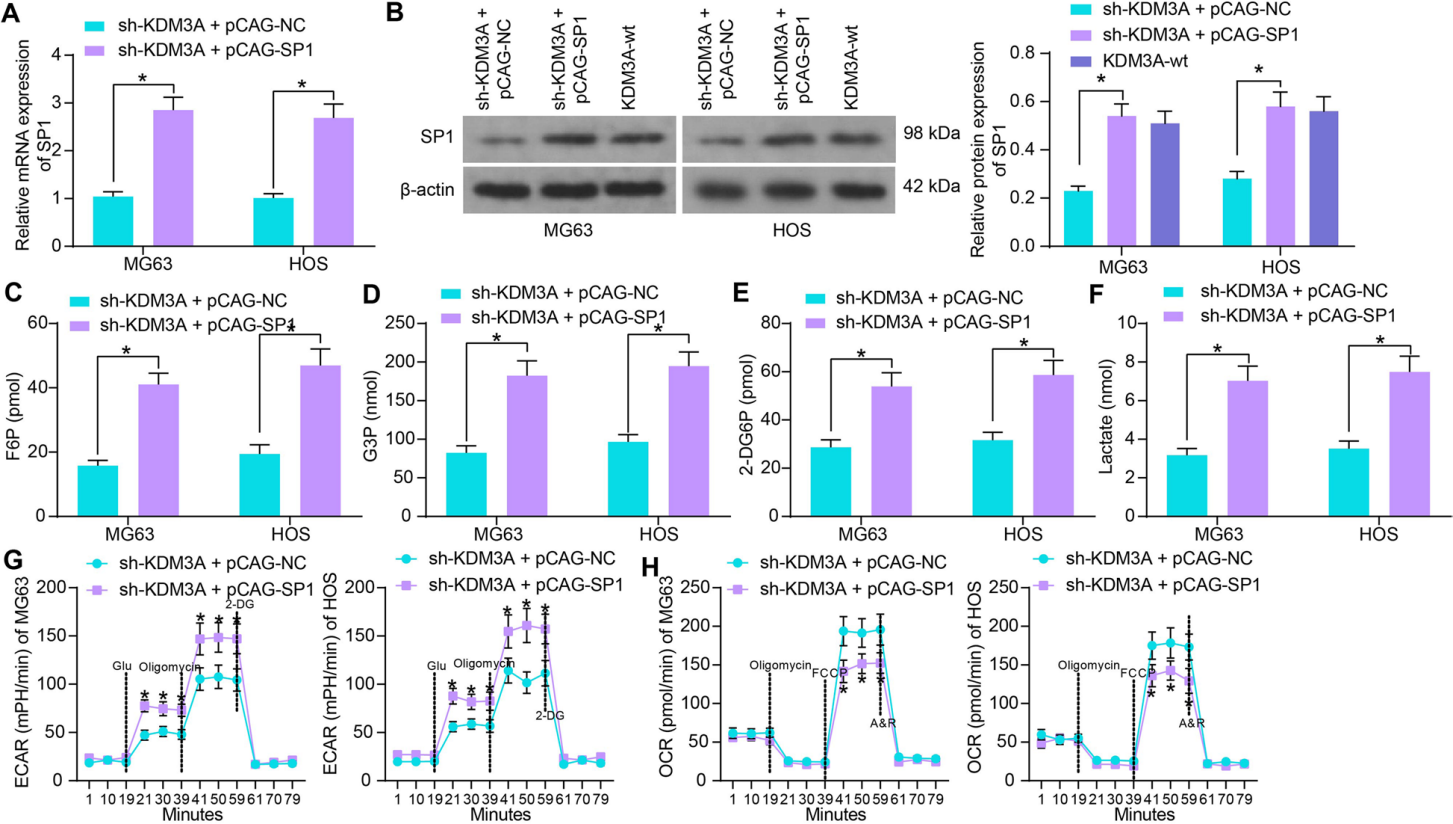
SP1 is essential for the KDM3A-mediated aerobic glycolysis in OS cells. **A** mRNA expression of SP1 in OS cells after further pCAG-SP1 transfection examined by RT-qPCR; **B** protein level of SP1 in sh-KDM3A- and pCAG-SP1-transfected OS cells, and in OS cells without KDM3A transfection (KDM3A-wt) determined by western blot analysis; **C**-**H** F6P production (**C**), G3P production (**D**), glucose uptake (**E**), lactate production (**F**), ECAR (**G**) and OCR (**H**) in OS cells after SP1 restoration. Three independent experiments were performed. Differences were analyzed by the two-way ANOVA (**A**-**H**) followed by Tukey’s test. *p < 0.05

Since KDM3A inhibition led to a decline in SP1 cells, SP1 overexpression plasmid pCAG-SP1 and the control pCAG-NC were additionally transfected into OS cells with stable sh-KDM3A transfection. Restoration of SP1 was detected in the OS cells (Fig. 5A). Moreover, the SP1 levels in sh-KDM3A- and pCAG-SP1-transfected OS cells, and in OS cells without KDM3A transfection (KDM3A-wt) were determined by western blot assay. It was observed that the pCAG-SP1-restored SP1 levels showed no significant difference from the endogenous SP1 levels in KDM3A-wt cells (Fig. 5B).

Thereafter, it was observed that overexpression of SP1 significantly elevated the production of F6P and G3P in cells (Fig. 5C, D). In addition, the glucose uptake and lactate production in OS cells were enhanced after SP1 restoration (Fig. 5E, F). Moreover, SP1 increased the ECAR (Fig. 5G) but reduced OCR (Fig. 5H) in cells. This body of evidence indicated that the aerobic glycolysis in OS cells suppressed by KDM3A silencing was recovered after SP1 restoration.

### SP1 plays crucial roles in KDM3A-mediated growth and metastasis of OS cells

**Fig. 6:**
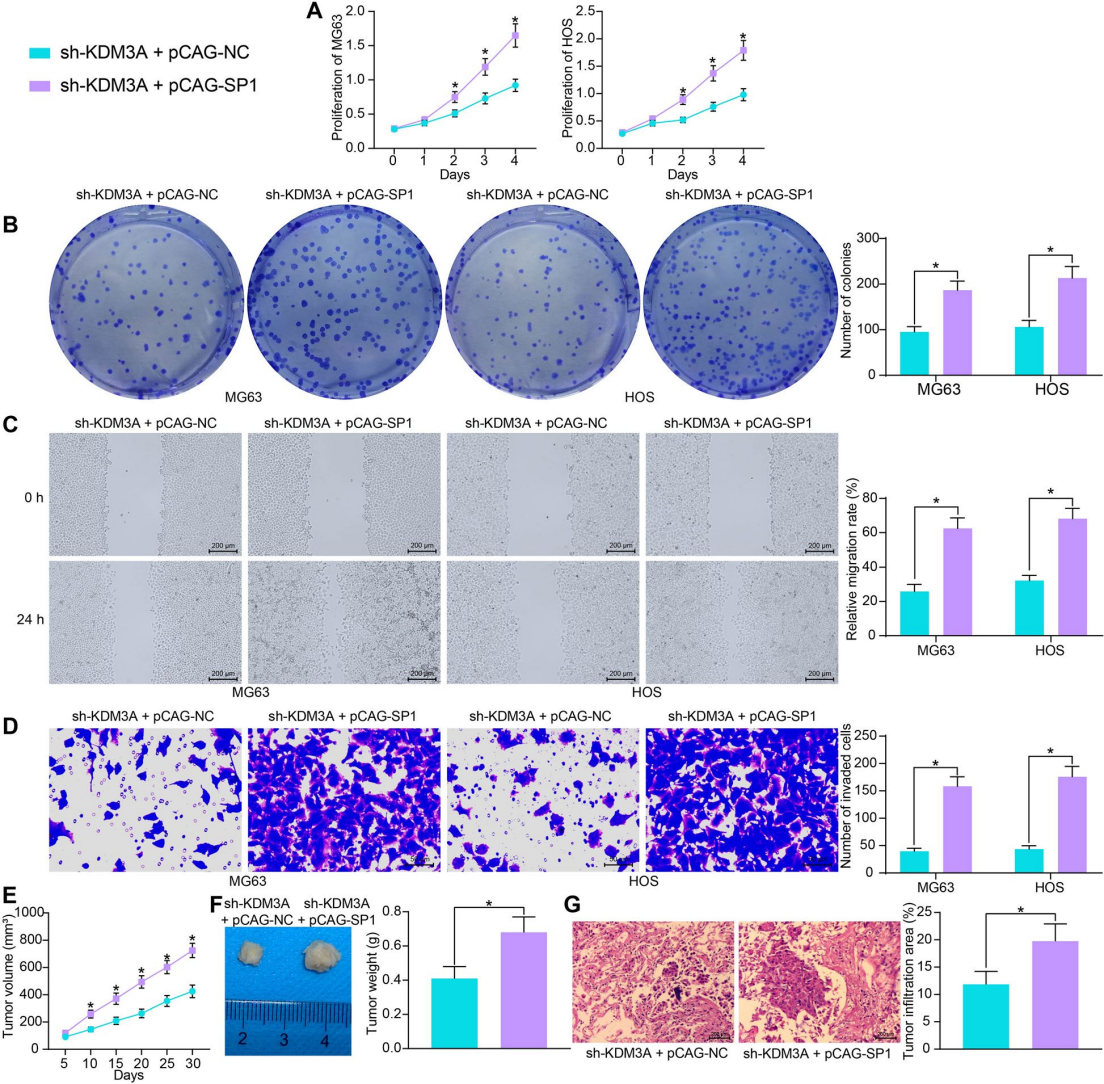
SP1 plays crucial roles in KDM3A-mediated growth and metastasis of OS cells. **A** proliferative activity of MG63 and HOS cells detected by the CCK-8 assay; **B** colony formation ability of MG63 and HOS cells examined by the colony formation assay; **C** migration of MG63 and HOS cells examined by the scratch test; **D** invasion of MG63 and HOS cells examined by the Transwell assay; **E** growth rate of xenograft tumors formed by HOS cells in nude mice; **F** weight of the xenograft tumors on day 30; **G** tumor infiltration in mouse lung tissues 45 days after tail vein injection of HOS cells examined by HE staining. For cellular experiments, three repetitions were performed; for animal experiments, *n* = 5 in each group. Data were analyzed by the unpaired *t* test (**F**-**G**) and two-way ANOVA (**A**-**E**) followed by Tukey’s test. *p < 0.05

In terms of cell behaviors, SP1 restoration significantly rescued the proliferative activity of OS cells (Fig. 6A). Likewise, the colony formation ability of the OS cells suppressed by sh-KDM3A was enhanced as well following SP1 overexpression (Fig. 6B). Moreover, overexpression of SP1 recovered the migration and invasion activities of OS cells (Fig. 6C, D). Similar results were reproduced in vivo. Upregulation of SP1 increased the growth of xenograft tumors formed by HOS cells (Fig. 6E, F). Also, the tumor metastasis in lung tissues, which was suppressed by sh-KDM3A, was increased after SP1 upregulation in HOS cells (Fig. 6G).

### KDM3A-SP1 mediates PFKFB4 expression

**Fig. 7:**
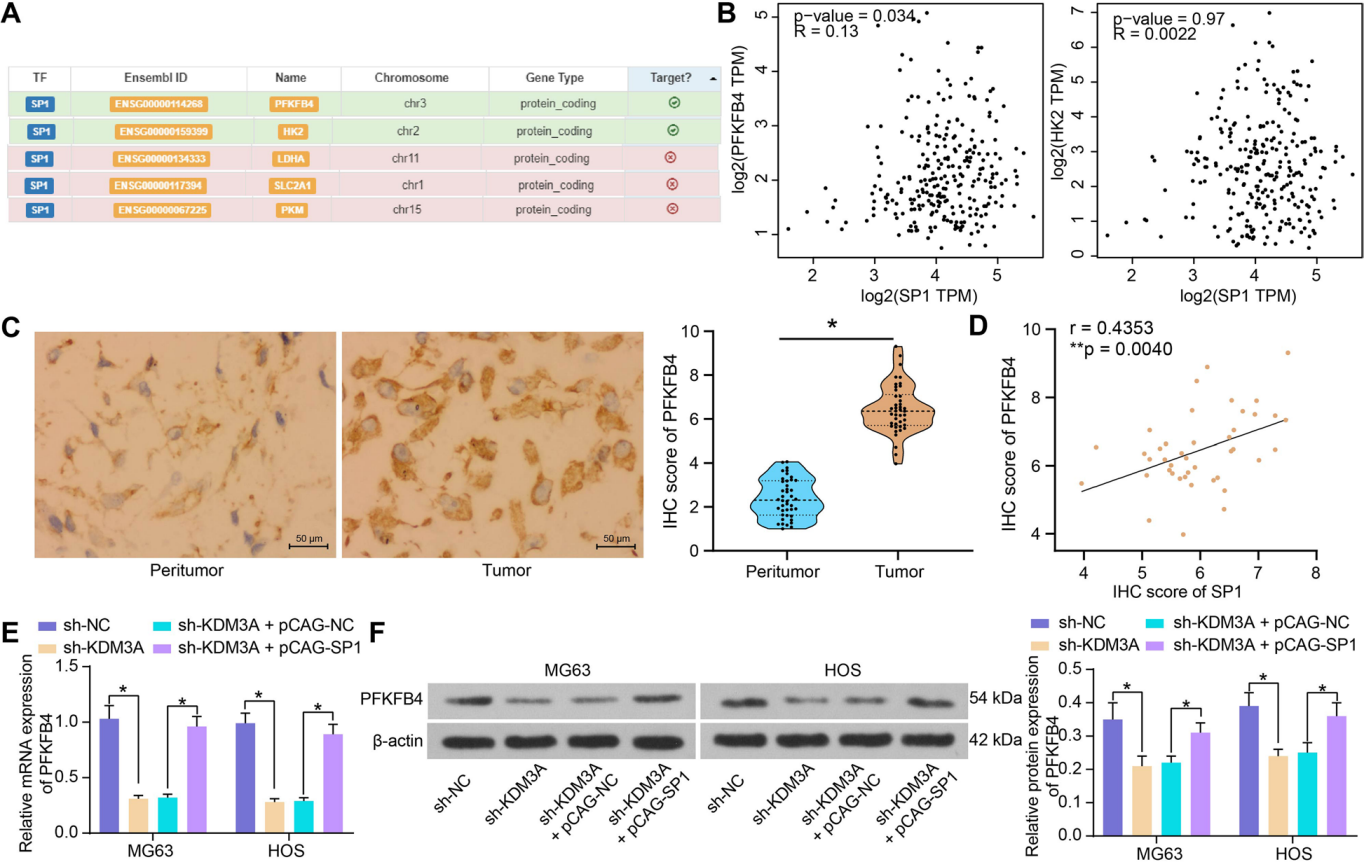
KDM3A-SP1 mediates PFKFB4 expression. **A** potential regulation of SP1 on several common key enzymes related to glycolysis predicted in the hTFtarget system; **B** correlations of SP1 with PFKGB4 and HK2 in sarcoma predicted in the GEPIA system; **C** IHC score of PFKFB4 in the collected tumor and peritumor bone tissue samples; **D** correlation between SP1 and PFKFB4 IHC scores in OS tissues; **E**-**F**, PFKFB4 mRNA (**E**) and protein (**F**) levels in OS cells transfected with sh-KDM3A/pCAG-SP1 determined by RT-qPCR and western blot analysis, respectively. Three independent experiments were performed. Differences were analyzed by the paired *t* test (**C**) and two-way ANOVA (**E**-**F**) followed by Tukey’s test. Correlation between variables was analyzed by the Pearson’s correlation analysis (**D**). *p < 0.05

SP1 acts as a transcriptional activator in cancer [24]. We then explored the correlation between SP1 and several common key enzymes related to glycolysis including PFKFB4, HK2, LDHA, SLC2A1 and PKM in hTFtarget (http://bioinfo.life.hust.edu.cn/hTFtarget/#!/). Interestingly, SP1 was predicted to have target relationships with PFKFB4 and HK2 (Fig. 7A). However, data in the Gene Expression Profiling Interactive Analysis (GEPIA; http://gepia.cancer-pku.cn/index.html) system suggested that SP1 had a significant positive correlation with PFKFB4 expression in sarcoma, but no significant correlation was found between SP1 and HK2 (Fig. 7B).

Thereafter, the IHC assay showed that PFKFB4 expression was significantly elevated in OS tissues (Fig. 7C), showing a positive correlation with SP1 (Fig. 7D). The PFKFB4 expression in OS cells was suppressed after KDM3A suppression but increased after SP1 restoration (Fig. 7E, F).

### SP1 targets PFKFB4 promoter to induce its transcription

**Fig. 8:**
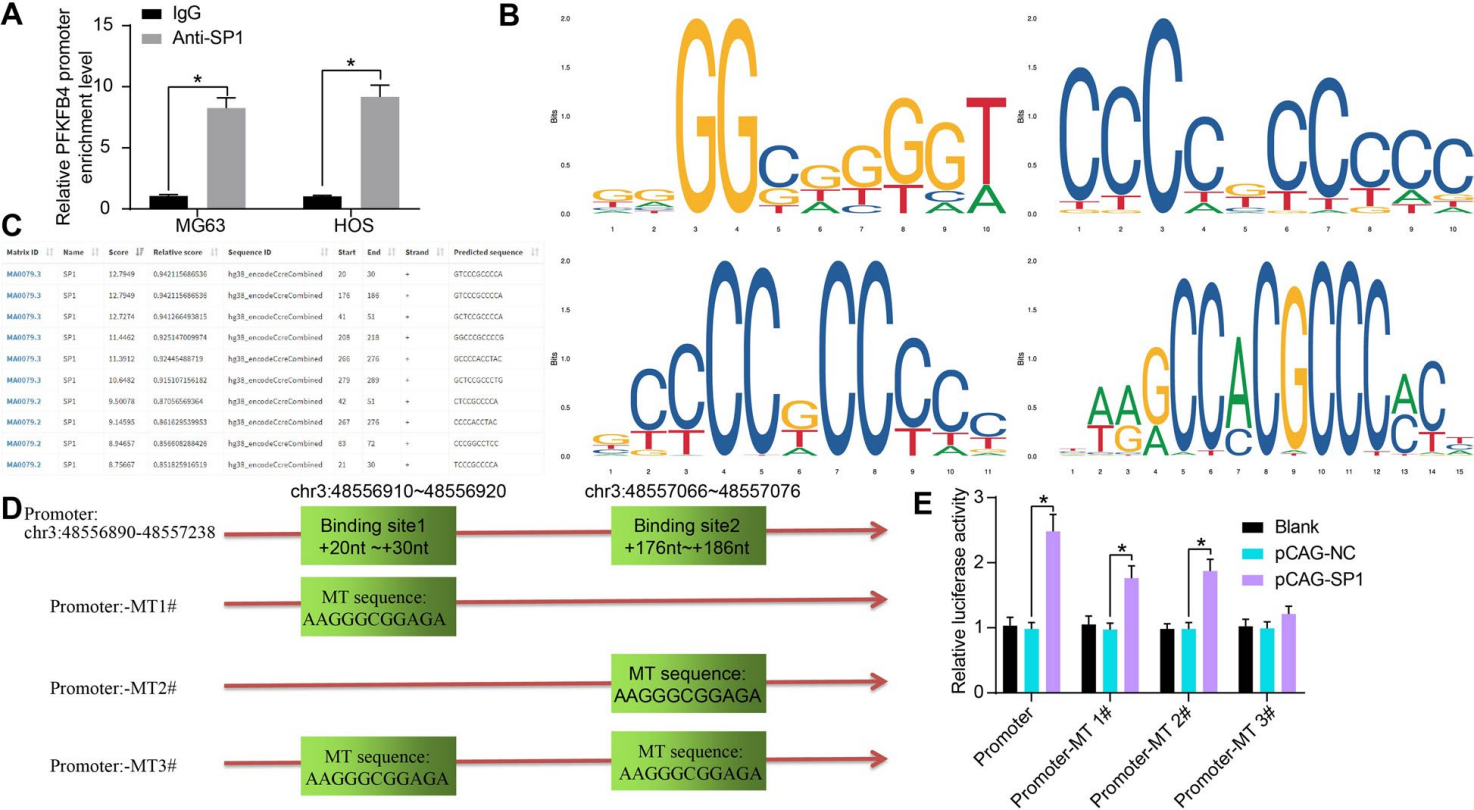
SP1 targets PFKFB4 promoter to induce its transcription. **A** PFKFB4 promoter sequence enriched by anti-SP1 in the ChIP-qPCR assay; **B** conservative transcriptional binding sequences of SP1; **C** PFKFB4 promoter sequences owning the binding relationship with SP1; **D** mutation of the PFKFB4 promoter sequence; **E** effect of SP1 overexpression on the transcriptional activity of the different PFKFB4 promoter sequences. Three independent experiments were performed. Differences were analyzed by the two-way ANOVA (**A**-**E**) followed by Tukey’s test. *p < 0.05

To confirm whether SP1 can directly bind to PFKFB4 promoter to induce its transcription, we first confirmed the binding relationship between SP1 and PFKFB4 promoter (chr3: 48,556,890–48,557,238) via the ChIP-qPCR assay (Fig. 8A).

To confirm the binding site between SP1 and the PFKFB4 promoter, we obtained the matrixes of the conservative transcriptional binding sequences of SP1 from the Jaspar system (Fig. 8B), and then analyzed the sequences within the PFKFB4 promoter owning the potential binding relationship with SP1 (Fig. 8C). Later, we observed that two SP1 binding sites with the highest scores were the same sequences that bound to the MA0079.3 matrix binding sequence of SP1 (Fig. 8C). Thereafter, the mutant sequences that could not bind to the MA0079.3 matrix sequence were designed, which were applied at the + 20 nt ~ + 30 nt sites (chr3: 48,556,910 ~ 48,556,920) and + 176 nt ~ + 186 nt sites (chr3: 48,557,066 ~ 48,557,076) of the PFKFB4 promoter sequence (Fig. 8D).

The luciferase reporter vectors based on the above sequences were constructed for luciferase assays (Fig. 8E). It was found that overexpression of SP1 in 293 T cells significantly increased the transcriptional activity of PFKFB4 promoter sequence. Mutation of either binding site (Promoter-MT 1# or Promoter-MT 2#) attenuated but not completely blocked the promoting effect of SP1 on the transcriptional activity of the PFKFB4 promoter. However, when both of the two binding sites were mutated (Promoter-MT 3#), overexpression of SP1 no longer significantly affected the transcriptional activity of the PFKFB4 promoter sequence.

### PFKFB4 overexpression promotes the growth and metastasis of OS cells

The function of PFKFB4 in OS remains unknown. The experiments above showed that KDM3A increased SP1 expression to promote PFKFB4 transcription. We therefore examined the role of PFKFB4 in OS cells to test its involvement in KDM3A-mediated oncogenic events.

**Fig. 9:**
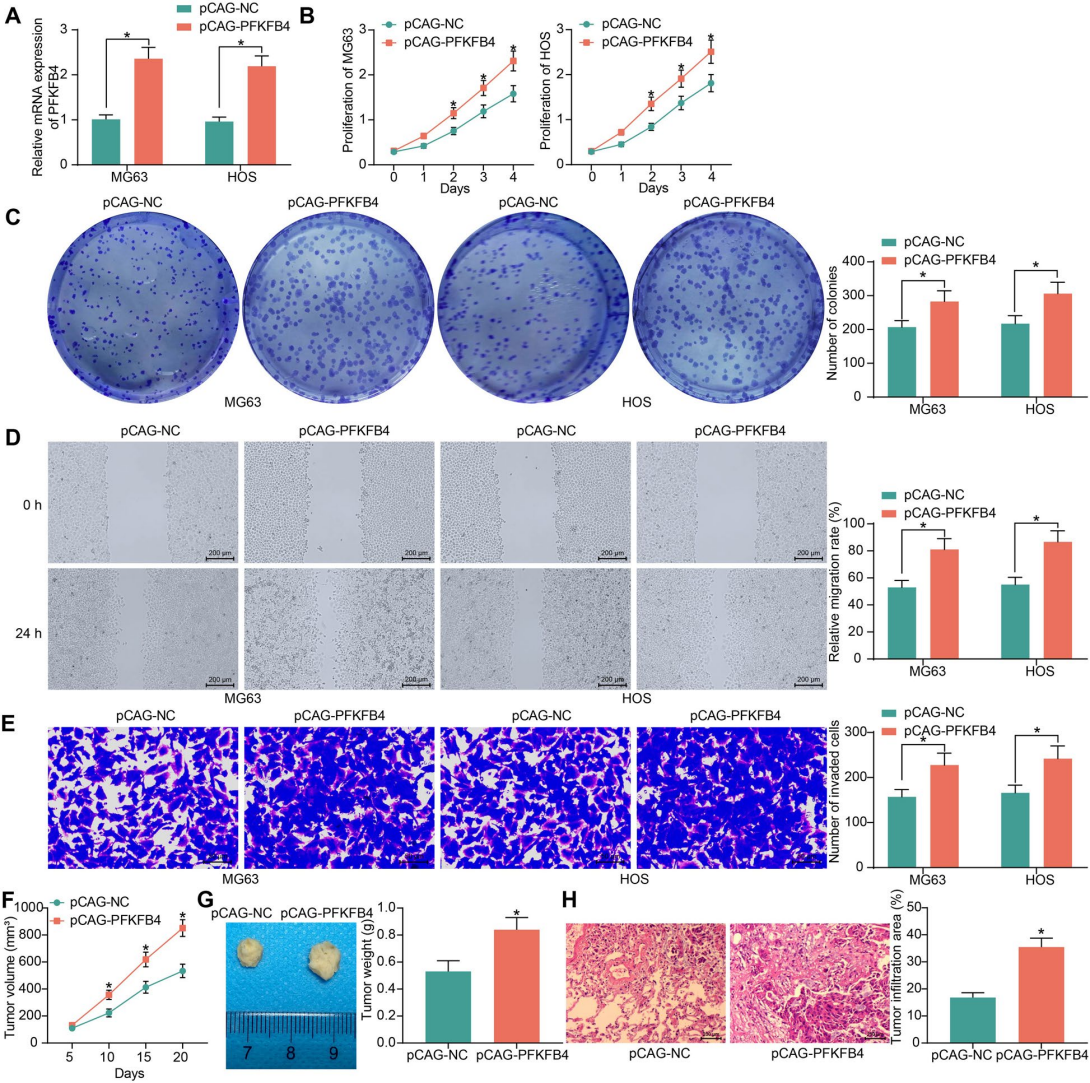
PFKFB4 overexpression promotes the growth and metastasis of OS cells. **A** transfection efficacy of pCAG-PFKFB4 in OS cells examined by RT-qPCR; **B** proliferative activity of MG63 and HOS cells detected by the CCK-8 assay; **C** colony formation ability of MG63 and HOS cells examined by the colony formation assay; **D** migration of MG63 and HOS cells examined by the scratch test; **E** invasion of MG63 and HOS cells examined by the Transwell assay; **F** growth rate of xenograft tumors formed by HOS cells in nude mice; **G** weight of the xenograft tumors on day 20; **H** tumor infiltration in mouse lung tissues 30 days after tail vein injection of HOS cells examined by HE staining. For cellular experiments, three repetitions were performed; for animal experiments, n = 5 in each group. Data were analyzed by the unpaired *t* test (**G**-**H**) and two-way ANOVA (**A**-**F**) followed by Tukey’s test. *p < 0.05

Overexpression of PFKFB4 was introduced in OS cells through the transfection of pCAG-PFKFB4 (Fig. 9A). The PFKFB4 overexpression significantly elevated proliferation (Fig. 9B) and colony formation (Fig. 9C) abilities of the OS cells. Moreover, it aggravated the migration and invasion potentials of the OS cells (Fig. 9D, E).

In vivo, overexpression of PFKFB4 increased the growth of xenograft tumors formed by HOS cells (Fig. 9F), and it led to increased tumor weight on day 20 after animal euthanasia (Fig. 9G). Also, injection of HOS cells overexpressing PFKFB4 through the tail vein significantly increased the tumor infiltration in mouse lung tissues (Fig. 9H).

### PFKFB4 enhances glycolytic flux in OS cells

**Fig. 10:**
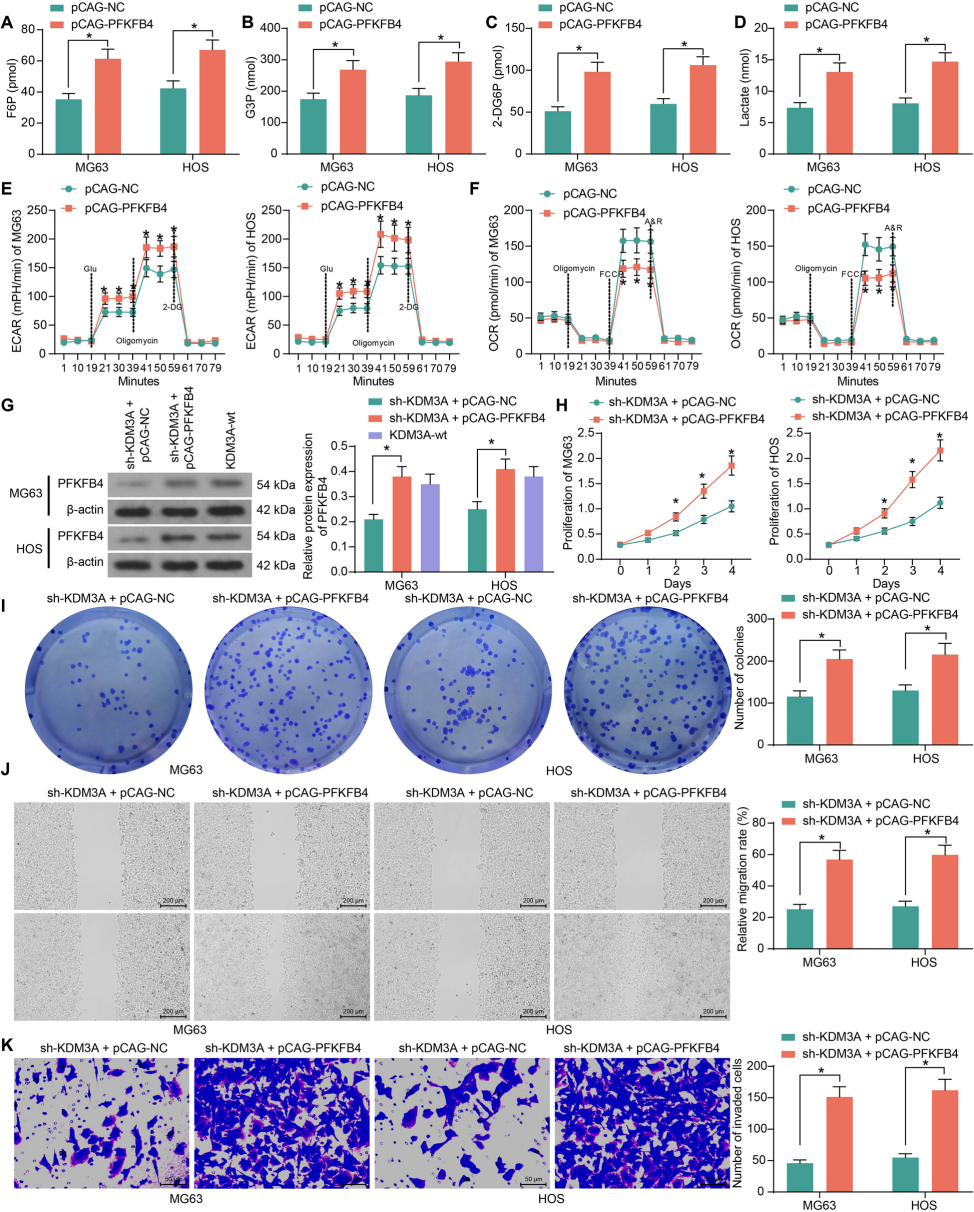
PFKFB4 enhances glycolytic flux in OS cells. F6P production (**A**), G3P production (**B**), glucose uptake (**C**), lactate production (**D**), ECAR (**E**) and OCR (**F**) in OS cells after PFKFB4 overexpression; **G** protein levels of PFKFB4 in cells determined by western blot analysis; **H** proliferative activity of MG63 and HOS cells detected by the CCK-8 assay; **I** colony formation ability of MG63 and HOS cells determined by the colony formation assay; **J** migration of MG63 and HOS cells examined by the scratch test; **K** invasion of MG63 and HOS cells examined by the Transwell assay. Three independent experiments were performed. Differences were analyzed by the two-way ANOVA (**A**-**K**) followed by Tukey’s test. *p < 0.05

As a glycolysis-related enzyme, the correlation of PFKFB4 with glycolytic activity in OS cells was examined. It was observed that overexpression of PFKFB4 in cells significantly elevated the production of F6P and G3P (Fig. 10A, B) and increased the levels of glucose uptake and lactate production (Fig. 10C, D). In line, PFKFB4 overexpression also increased the ECAR (Fig. 10E) but suppressed OCR of the OS cells (Fig. 10F). This evidence suggested that upregulation of PFKFB4 is responsible for the oncogenic and pro-glycolytic roles of KDM3A in OS.

In addition, to examine the correlation between KDM3A and PFKFB4, pCAG-PFKFB4 was transfected into OS cells stably transfected with sh-KDM3A. Western blot assays showed that pCAG-PFKFB4 restored the PFKFB4 levels in cells. Although the restored PFKFB4 expression was slightly higher than the endogenous PFKFB4 expression in KDM3A-wt cells, there was no significant difference (Fig. 10G). The PFKFB4 restoration significantly rescued the proliferation, colony formation, migration, and invasion of OS cells that were blocked by sh-KDM3A (Fig. 10H-K).

## Discussion

The aerobic glycolysis is essential to meet the metabolic requirements of cancer cell proliferation [9]. This is also true for OS cells, since suppression of the glycolytic process has been correlated with blocked tumor growth, survival, and metastasis [25]. In this research, we confirmed that the KDM3A-SP1 axis-mediated PFKFB4 upregulation promoted aerobic glycolysis in OS and augmented tumor cell growth and metastasis.

Epigenetic modifiers have aroused increasing concerns regarding their key roles and frequent involvements in tumorigenesis [26]. KDM3A has been summarized to be aberrantly expressed and associated with cancer development through its potent regulation on gene expression by removing H3K9me1/me2 [15]. KDM3A can regulate expression of oncogenes by binding to androgen receptor [27, 28], estrogen receptor [29], hypoxia-inducible factor (HIF)-1α [30, 31], or modulating downstream pathways such as the Wnt/β-catenin signaling [32] and glycolytic pathways [15]. In a study by Parrish et al., KDM3A was first identified to be highly expressed in Ewing Sarcoma, and its depletion led to H3K9me2 upregulation and the subsequent downregulation of oncogenes and impaired tumorigenesis [33]. Their following study in 2017 further demonstrated that KDM3A positively regulated metastasis-related genes in Ewing Sarcoma [16]. Based on this research, KDM3A was then suggested as a candidate therapeutic target for Ewing Sarcoma treatment [34]. However, compared to the known effect of KDM3A in Ewing Sarcoma, less is known regarding its function in the more common type of bone tumor OS. In the present study, we validated that KDM3A was highly expressed in the OS tumor tissues and cells, and it was correlated with disease prognosis in patients. A cDNA microarray analysis by Cho et al. suggested that KDM3A is highly expressed in multiple cancers including OS [35]. Similarly, upregulation of KDM3A was found to be correlated with poor prognosis in other malignancies such as pancreatic cancer [36] and colorectal cancer [37]. A recent study by Li et al. suggested that HIF-3α was significantly upregulated in OS and it activated the KDM3A transcription under hypoxic conditions [38]. This may represent one of the mechanisms for aberrant KDM3A overexpression in OS. Importantly, we further found that KDM3A was crucial for the maintenance of the growth and metastasis of OS cells as well as the aerobic glycolysis in cells. KDM3A has been reported to demethylate H3K9me2 on the promoter of glycolytic gene PGK, and it cooperated with HIF-1α to induce aerobic glycolysis in urinary bladder cancer [39]. Intriguingly, KDM3A has been reported to demethylate monomethylated lysine (K) 224 of PGC-1α and lead to PGC-1α-dependent mitochondrial biogenesis under normoxic conditions [40], while mitochondrial biogenesis is often upregulated in cancers and plays a central and multi-functional role in malignant tumor progression [41]. Moreover, mitochondrial respiration defects were initially considered as an underlying basis for aerobic glycolysis and cancer; however, they are recognized more and more not as a general character of all tumors or less selected during tumor evolution [41]. In this report, we only performed loss-of-function assays of KDM3A to examine its relevance to cancer cell development. Overexpression of KDM3A, not surprisingly, has been correlated with aggravation of cancers [37, 42].

As KDM3A usually activates gene transcription by removing the transcriptional repressive markers H3K9me1/me2 [15], integrated bioinformatics analyses were performed on the KDM3A-positively-related genes, and SP1 was identified as a target of KDM3A in OS. KDM3A mediated SP1 upregulation by demethylating H3K9me2. It has been reported that there are over 12,000 SP binding sites within the human genome, and SP1 not only maintains basal transcription, but also regulates transcription of a large number of cellular genes [18]. Due to its frequent implication and close correlation with cell growth, differentiation, and carcinogenesis, SP1 has been suggested as a candidate long-standing target in cancer therapy [43]. The clear oncogenic role of SP1 has been observed in multiple diseases, such as ovarian cancer [44], prostate cancer [45], breast cancer [46], Ewing sarcoma [47], and OS [48]. The subsequent rescue experiments in this work suggested that restoration of SP1 re-enhanced the growth and metastasis of OS cells and recovered the glycolytic flux in cells suppressed by knockdown of KDM3A. ΔNp63α and SP1-mediated monocarboxylate transporter 4 has been observed to be correlated with the aerobic glycolysis-preference subtype of non-small cell lung cancer [49]. Likewise, cooperation of HIF-1α and SP1-mediated CD147 led to increased glycolysis and tumor progression in epithelial solid tumors [50]. Here, we observed that SP1 is required for the glycolytic activity in OS. However, the downstream molecules remained to be explored.

To this end, the glycolysis-related enzymes that were possibly mediated by SP1 were explored, and PFKFB4 was identified. SP1 bound to the PFKFB4 promoter for transcriptional activation. PFKFB4 encodes 6-phosphofructo-2-kinase/fructose-2,6-bisphosphatase-4, a bifunctional metabolic enzyme that synthesizes F2,6-BP, a crucial sugar-phosphate metabolite that stimulates glycolysis [51, 52]. Not surprisingly, PFKFB4-mediated glucose metabolism promoted cancer cell proliferation and maintenance of stemness of stem cells [51, 53–55]. Here, we found that PFKFB4 was highly expressed in the OS tissues and suppressed by KDM3A silencing, whereas it was upregulated by SP1 in the OS cell lines. The subsequent functional experiments showed that upregulation of PFKFB4 significantly promoted growth and metastasis of OS cells, which might be attributed to the dramatically increased glycolytic flux in cells. This body of evidence elucidated that PFKFB4 upregulation was responsible for the pro-glycolytic and pro-tumorigenic roles of KDM3A and SP1 in OS.

## Conclusion

**Fig. 11:**
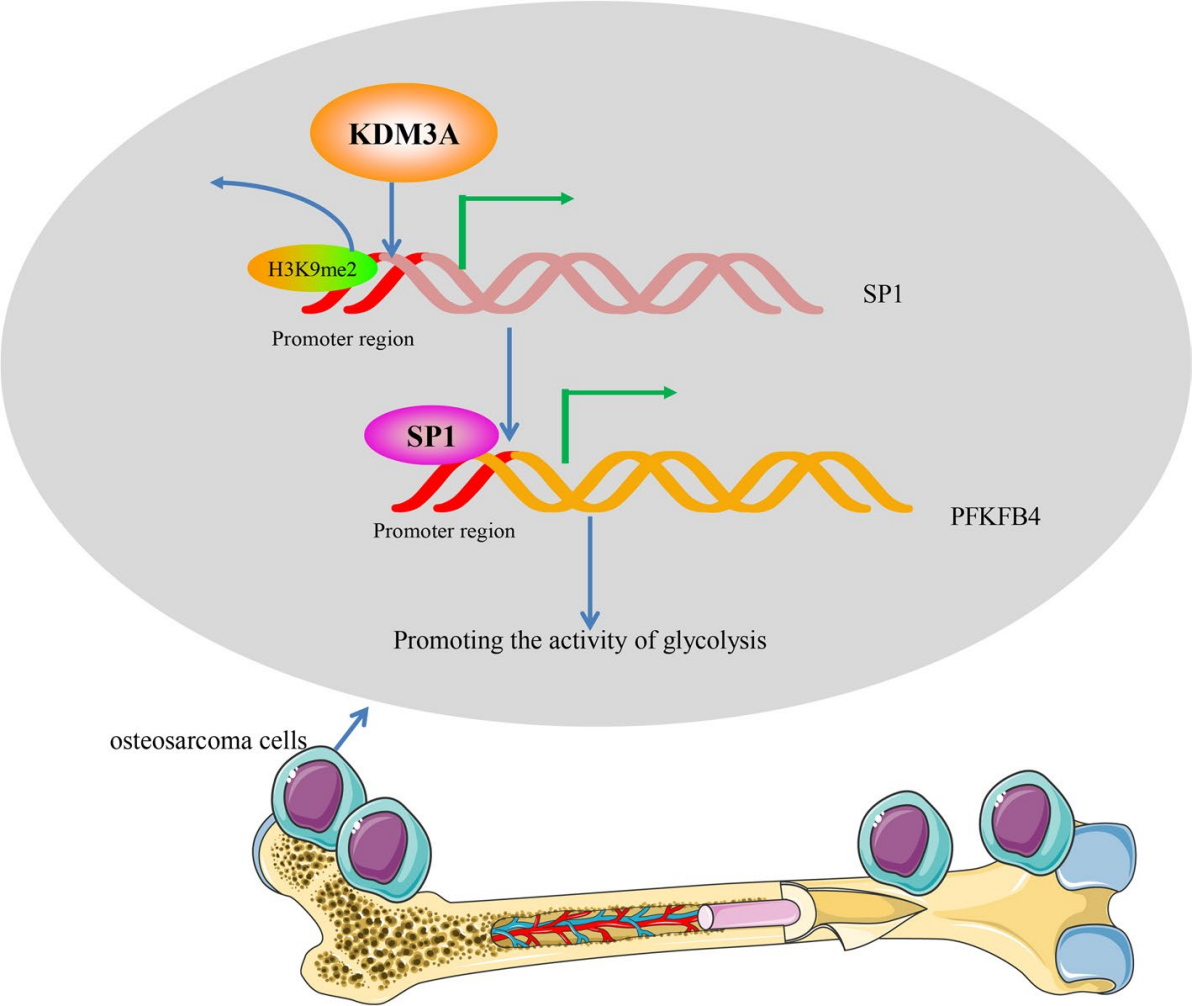
Graphical presentation for the molecular mechanism. KDM3A is upregulated in OS and promotes the transcription of SP1 through demethylating H3K9me2. SP1 further activates the transcription of PFKFB4 to promote glycolytic activity in OS cells and induce tumor progression

Taken together, the present study demonstrates that the histone modifier KDM3A epigenetically activates SP1 transcription, which further activates the glycolysis-related PFKFB4 in OS, consequently leading to tumor cell growth and metastasis (Fig. 11). This work may provide novel thoughts into the management of OS that the KDM3A-SP1-PFKFB4 axis may serve as a novel target for OS therapy.

## Supplementary Information

**Additional file 1.**

## Data Availability

All the data generated or analyzed during this study are included in this published article.

## Abbreviations

ANOVA: Analysis of variance
CCK-8: Cell counting kit-8
ChIP: Chromatin immunoprecipitation
ECR: Extracellular acidification rate
ELISA: Enzyme-linked immunosorbent assay
F6P: Fructose-6-phosphate
FBS: Fetal bovine serum
G3P: Glyceraldehyde-3-phosphate
GEPIA: Gene Expression Profiling Interactive Analysis
H3K9me1/me2: Mono- and di-methylated histone H3 lysine 9
HE staining: Hematoxylin and eosin staining
HRP: Horseradish peroxidase
IHC: Immunohistochemistry
KEGG: Kyoto Encyclopedia of Genes and Genomes
KDM3A: Lysine-specific histone demethylase 3A
mean ± SD: Mean ± standard deviation
MT: Mutant type
NC: Negative control
OCR: Oxygen consumption rate
OD: Optical density
OS: Osteosarcoma
PBS: Phosphate-buffered saline
PFA: Paraformaldehyde
PFKFB4: 6-phosphofructo-2-kinase/fructose-2,6-biphosphatase 4
RIPA: Radio-immunoprecipitation assay
RT-qPCR: Reverse transcription-quantitative polymerase chain reaction
shRNA: Short hairpin RNA
SIN3A: SIN3 transcription regulator family member A
SP1: Sp1 transcription factor
TCGA: The Cancer Genome Atlas
WT: Wild type
