# Supplementary figures and images for: KDM3A-mediated SP1 activates PFKFB4 transcription to promote aerobic glycolysis in osteosarcoma and augment tumor development
Source: BMC Cancer. 2022 May 19;22:562. doi: 10.1186/s12885-022-09636-8 (PMC9118730; doi:10.1186/s12885-022-09636-8)

Fig 1E

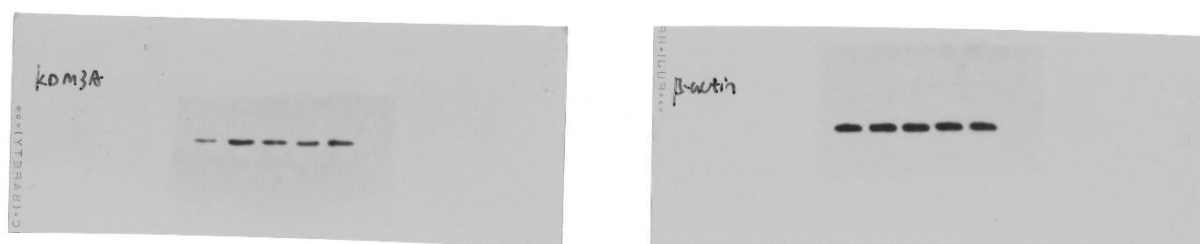

Fig 4G

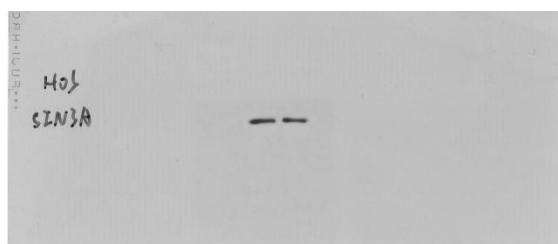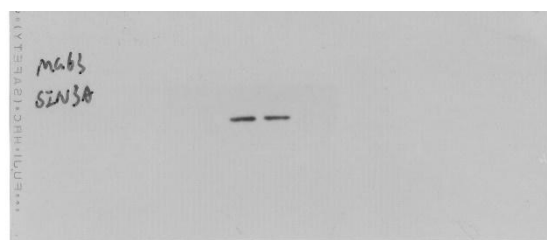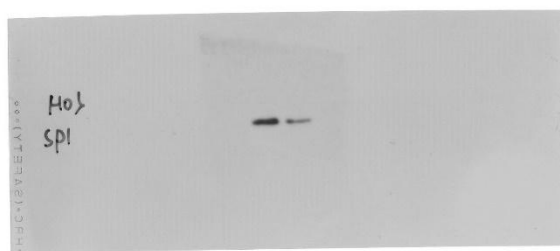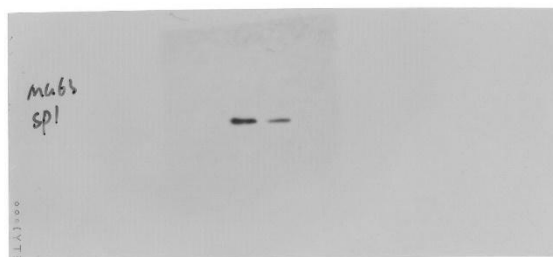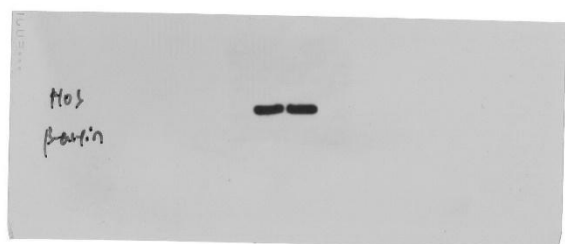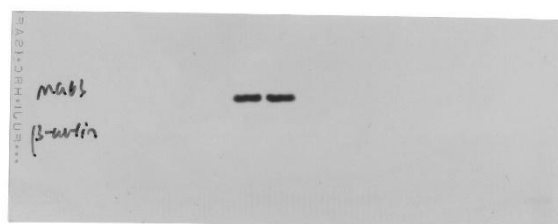

Fig 5B

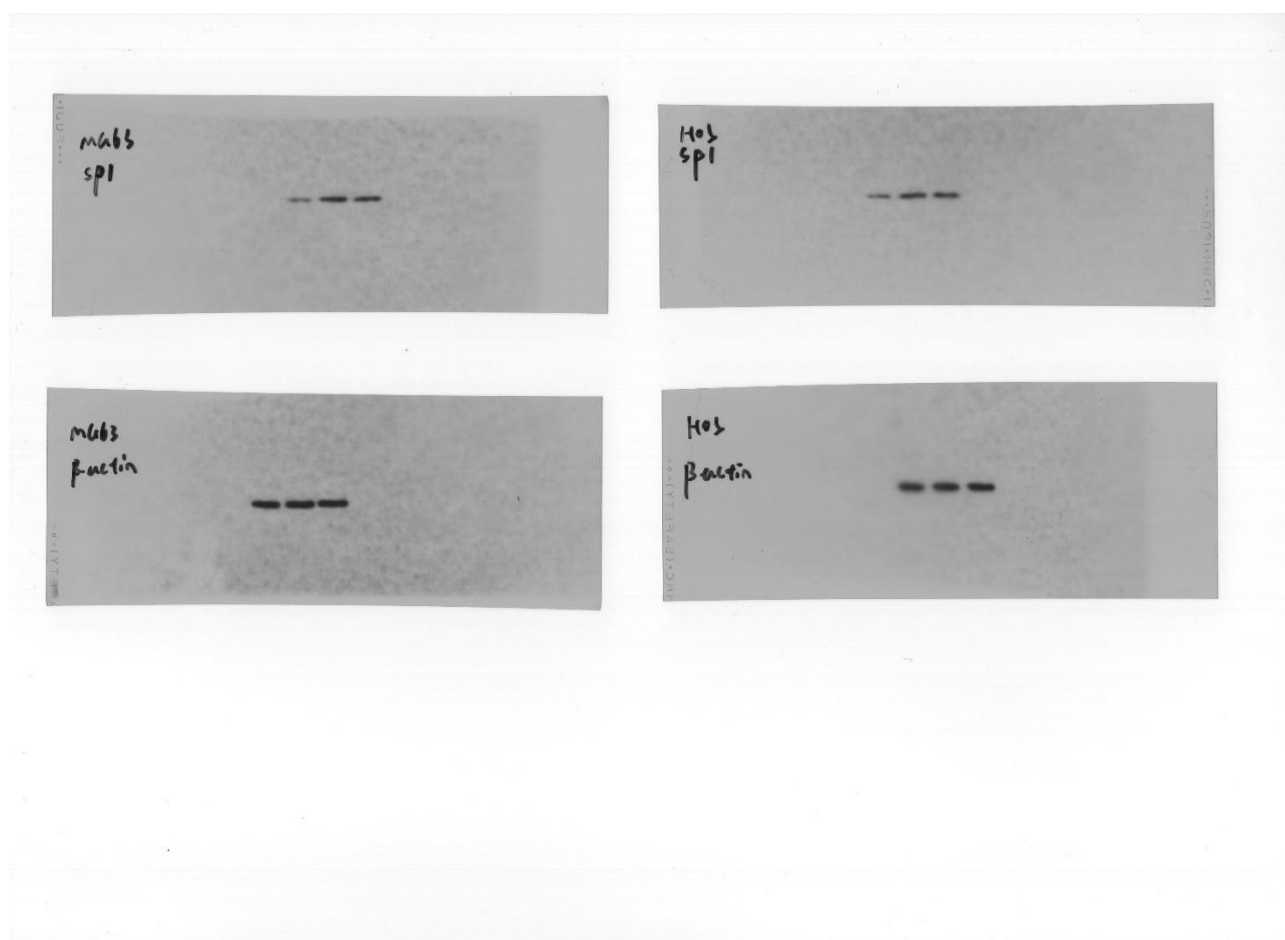

Fig 7F

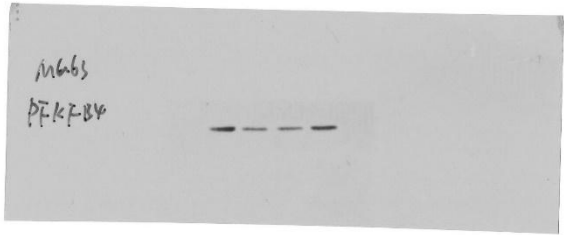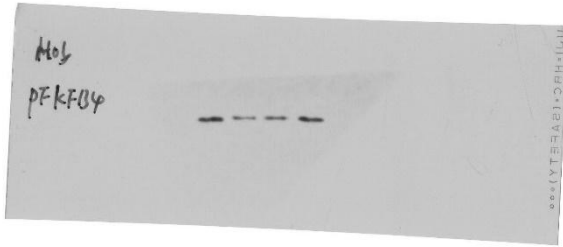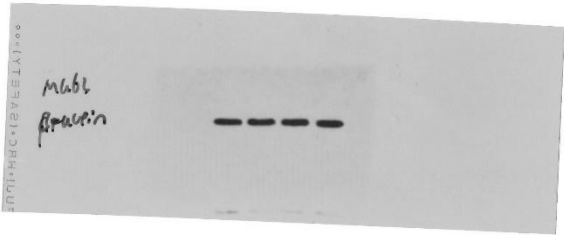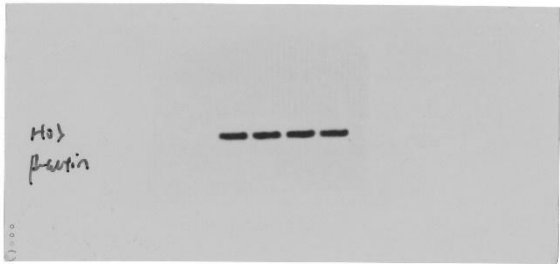

Fig 10G

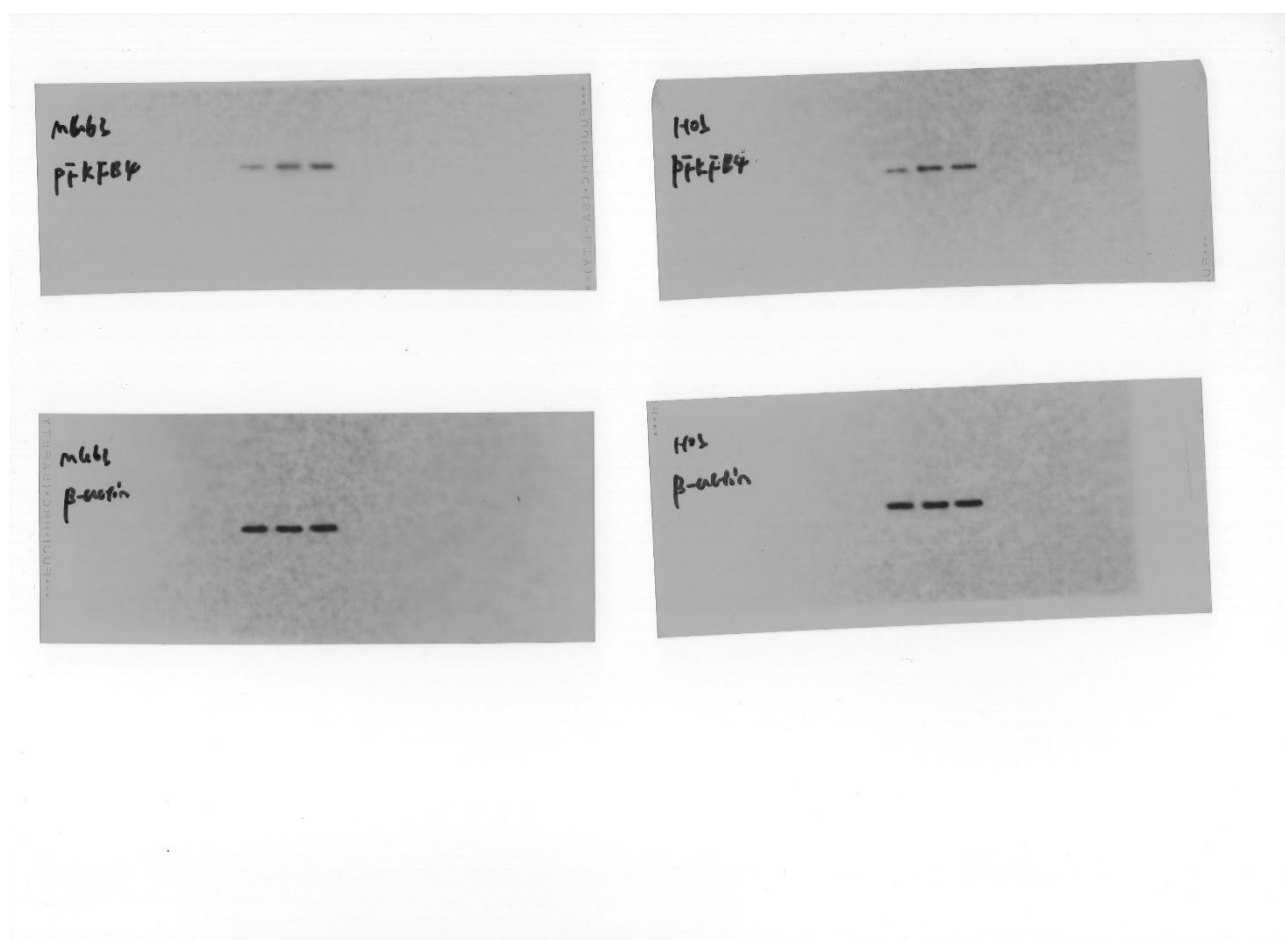

Supplement: Supplementary file 1 — Additional file 1. [file 12885_2022_9636_MOESM1_ESM.pdf]
